# 5-Benz­yloxy-3-methyl-1-tosyl-1*H*-indole

**DOI:** 10.1107/S1600536813014001

**Published:** 2013-05-31

**Authors:** Gustavo Pozza Silveira, Allen G. Oliver, Bruce C. Noll

**Affiliations:** a Departamento de Química Orgânica, Instituto de Química, Universidade Federal do Rio Grande do Sul, Porto Alegre/RS, 91501-970, Brazil; b Department of Chemistry and Biochemistry, University of Notre Dame, Notre Dame, IN 46556, USA; c Bruker ACS Inc., 5465 East Cheryl Parkway, Madison, WI 53711, USA

## Abstract

The title compound, C_23_H_21_NO_3_S, represents one of the few examples of a 5-substituted indole with a toluene­sulfonyl group bonded to the N atom. The benzyl group adopts a synclinal geometry with respect to the indole ring [dihedral angle = 59.95 (4)°], while the tolyl ring is oriented close to perpendicular to the indole ring, making a dihedral angle of 81.85 (3)°. The indole N atom exhibits a slight pyramidalization.

## Related literature

For background to physostigmine and related marine natural products, see: Marino *et al.* (1989 ▶, 1992 ▶). For recent, related structural and synthetic studies, see: Pozza Silveira *et al.* (2012 ▶); Silveira & Marino (2013 ▶). For related compounds, see: Xiong *et al.* (2001 ▶); Witulski *et al.* (2000 ▶). For reference structural data see: Allen *et al.* (1995 ▶).
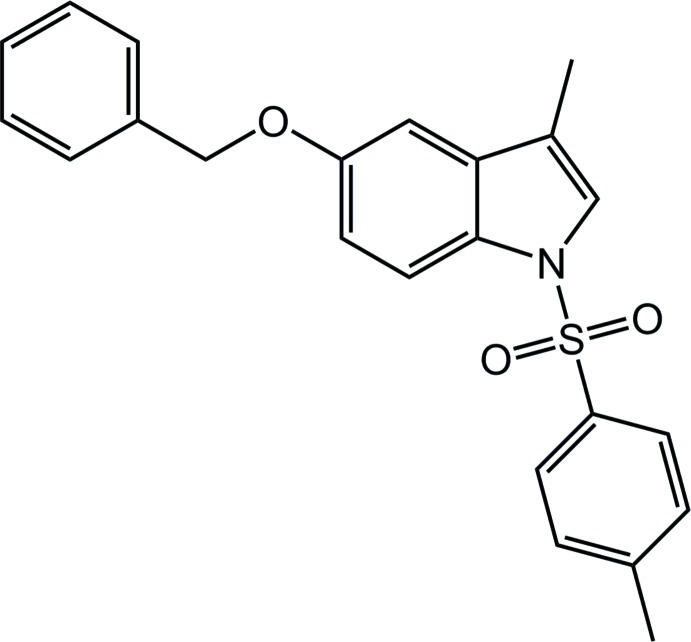


## Experimental

### 

#### Crystal data

C_23_H_21_NO_3_S
*M* *_r_* = 391.47
Monoclinic, 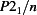

*a* = 8.317 (3) Å
*b* = 15.601 (6) Å
*c* = 14.752 (5) Å
β = 90.884 (11)°
*V* = 1914.1 (12) Å^3^
*Z* = 4
Mo *K*α radiation
μ = 0.19 mm^−1^
*T* = 100 K
0.39 × 0.33 × 0.15 mm

#### Data collection

Bruker APEXII diffractometer
Absorption correction: multi-scan (*SADABS*; Bruker, 2008 ▶) *T*_min_ = 0.636, *T*_max_ = 0.746
30660 measured reflections
6415 independent reflections
5550 reflections with *I* > 2σ(*I*)
*R*_int_ = 0.026

#### Refinement

*R*[*F* ^2^ > 2σ(*F* ^2^)] = 0.037
*wR*(*F* ^2^) = 0.102
*S* = 1.04
6415 reflections
255 parameters
H-atom parameters constrained
Δρ_max_ = 0.59 e Å^−3^
Δρ_min_ = −0.42 e Å^−3^

### 

Data collection: *APEX2* (Bruker, 2008 ▶); cell refinement: *SAINT* (Bruker, 2008 ▶); data reduction: *SAINT*; program(s) used to solve structure: *SHELXS97* (Sheldrick, 2008 ▶); program(s) used to refine structure: *SHELXL2012* (Sheldrick, 2008 ▶); molecular graphics: *XP* in *SHELXTL* (Sheldrick, 2008 ▶); software used to prepare material for publication: *publCIF* (Westrip, 2010 ▶).

## Supplementary Material

Crystal structure: contains datablock(s) I, global. DOI: 10.1107/S1600536813014001/kj2226sup1.cif

Structure factors: contains datablock(s) I. DOI: 10.1107/S1600536813014001/kj2226Isup2.hkl

Supplementary material file. DOI: 10.1107/S1600536813014001/kj2226Isup3.cml

Additional supplementary materials:  crystallographic information; 3D view; checkCIF report

## Figures and Tables

**Fig. 1.**
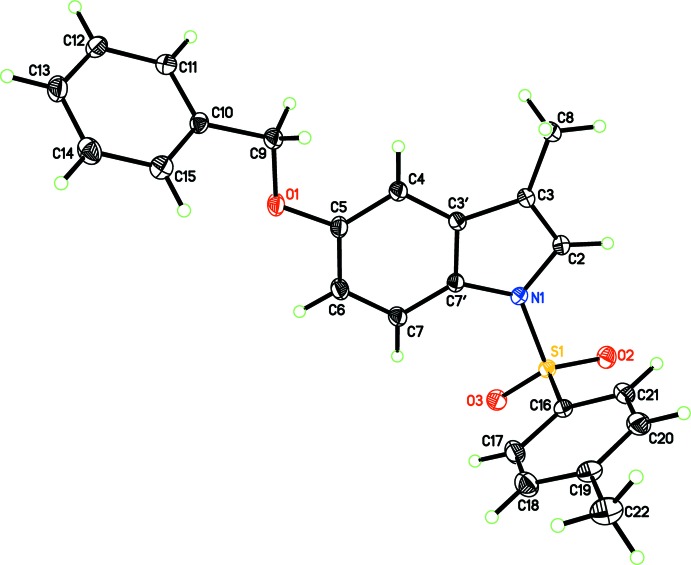
Labelling scheme for the title compound. Displacement ellipsoids depicted at 50% probability level. The labeling scheme follows the convention for indoles.

## supplementary crystallographic information

### Comment

Substituted indoles serve as unique precursors for medicinally important physostigmine alkaloids, which are anticholinesterases and miotics. This alkaloid skeleton has been found in marine alkaloids from *Broyoza Flustra foliacea* in the flustramines (Marino *et al.*, 1989) The literature presents a great number of enantiocontrolled syntheses for the natural and unnatural physostigmine (Marino *et al.*, 1992) Our interest in physostigmine emanated from our recent asymmetric synthesis of naphthyl lactams (Silveira & Marino, 2013) using chiral vinyl sulfilimines (Pozza Silveira *et al.*, 2012). Herein we report 5-(benzyloxy)-3-methyl-1-tosyl-1*H*-indole as a potential physostigmine precursor.

The title compound, C_23_H_21_NO_3_S, exhibits no unusual structural features and represents one of the few examples of an indole with a toluenesulfonyl bonded to the nitrogen and an oxygen bridging moiety bonded in the 5-position on the indole ring. The two other examples are 5-cyano-2-methoxy-4-trifluoromethyl-6-[3'-(*N*-toluenesulfonyl-5'-µethoxyindolyl)]pyridine (Xiong *et al.*, 2001) and 1-(4-methylphenyl)sulfonyl-3-((*E*)-2-(benzyl((4-methylphenyl)sulfonyl)αmino)ethenyl)-5-methoxy-1*H*-indole (Witulski *et al.*, 2000). In both of these examples the 5-position is occupied by a methoxy group.

Bond distances and angles are within normal, acceptable ranges (Allen *et al.* 1995). The benzyl moiety adopts a *syn*-clinal geometry with respect to the indole ring (interplanar angle = 59.95 (4)°), while the tolyl ring is oriented close to perpendicular to the indole ring (interplanar angle = 81.85 (3)°). The complement of this angle, 108.15°, is close to the N1—S1—C16 angle (104.19 (5)°). The difference is a consequence of a slight pyramidalization of the indolic nitrogen.

### Experimental

To a stirred solution of dimsylsodium [prepared from 110 mg (2.75 mmol) of NaH 60% dispersion in mineral oil and dimethylsulfoxide dry (0.58 ml) at 338 to 343 K until H_2_ is no longer evolved] was added a solution of 5-benzyloxy-3-methylindole (325 mg, 1.37 mmol) in dry THF (0.9 ml) under ice cooling. After stirring at room temperature for 1 h a solution of 4-methylbenzenesulfonyl chloride (225 mg, 1.18 mmol) in THF (0.9 ml) was added to this mixture at 273 K. After being stirred at room temperature for 16 h, the reaction product was poured into water and extracted with ethyl acetate. The extract was washed with water, dried over Na_2_SO_4_, and evaporated. The remaining residue was recrystallized from ethyl acetate/hexanes to give 482 mg of the desired product as white needle crystals (90%): mp 402 to 403 K. A suitably sized, block-like crystal was cut from a larger, columnar crystal for the diffraction study.

### Refinement

Hydrogen atoms were included in geometrically calculated positions. C—H distances were constrained to 0.95 Å for aromatic and 0.98 - 0.99 Å for aliphatic hydrogen atoms. Methyl hydrogen atoms were refined with thermal parameters restrained to *U*_iso_H = 1.5 × *U*_eq_C and all other hydrogen atoms = 1.2 × *U*_eq_C of the carbon to which they are bonded.

### Crystal data

**Table:**

C_23_H_21_NO_3_S	*D*_x_ = 1.358 Mg m^−^^3^
*M**_r_* = 391.47	Melting point: 402 K
Monoclinic, *P*2_1_/*n*	Mo *K*α radiation, λ = 0.71073 Å
*a* = 8.317 (3) Å	Cell parameters from 9980 reflections
*b* = 15.601 (6) Å	θ = 2.6–31.6°
*c* = 14.752 (5) Å	µ = 0.19 mm^−^^1^
β = 90.884 (11)°	*T* = 100 K
*V* = 1914.1 (12) Å^3^	Block, colourless
*Z* = 4	0.39 × 0.33 × 0.15 mm
*F*(000) = 824	

### Data collection

**Table:**

Bruker APEXII diffractometer	6415 independent reflections
Radiation source: fine-focus sealed tube	5550 reflections with *I* > 2σ(*I*)
Graphite monochromator	*R*_int_ = 0.026
Detector resolution: 8.33 pixels mm^-1^	θ_max_ = 31.6°, θ_min_ = 1.9°
combination of ω and φ–scans	*h* = −10→12
Absorption correction: multi-scan (*SADABS*; Bruker, 2008)	*k* = −22→22
*T*_min_ = 0.636, *T*_max_ = 0.746	*l* = −21→21
30660 measured reflections	

### Refinement

**Table:**

Refinement on *F*^2^	Primary atom site location: structure-invariant direct methods
Least-squares matrix: full	Secondary atom site location: difference Fourier map
*R*[*F*^2^ > 2σ(*F*^2^)] = 0.037	Hydrogen site location: inferred from neighbouring sites
*wR*(*F*^2^) = 0.102	H-atom parameters constrained
*S* = 1.04	*w* = 1/[σ^2^(*F*_o_^2^) + (0.0536*P*)^2^ + 0.775*P*] where *P* = (*F*_o_^2^ + 2*F*_c_^2^)/3
6415 reflections	(Δ/σ)_max_ = 0.001
255 parameters	Δρ_max_ = 0.59 e Å^−^^3^
0 restraints	Δρ_min_ = −0.42 e Å^−^^3^

### Special details

**Table:**

Geometry. All e.s.d.'s (except the e.s.d. in the dihedral angle between two l.s. planes) are estimated using the full covariance matrix. The cell e.s.d.'s are taken into account individually in the estimation of e.s.d.'s in distances, angles and torsion angles; correlations between e.s.d.'s in cell parameters are only used when they are defined by crystal symmetry. An approximate (isotropic) treatment of cell e.s.d.'s is used for estimating e.s.d.'s involving l.s. planes.

### Fractional atomic coordinates and isotropic or equivalent isotropic displacement parameters (Å^2^)

**Table:**

	*x*	*y*	*z*	*U*_iso_*/*U*_eq_	
N1	0.84082 (10)	0.26302 (5)	0.60391 (6)	0.01478 (15)	
O1	0.35145 (9)	0.40007 (6)	0.39442 (5)	0.02043 (16)	
S1	0.98068 (3)	0.19050 (2)	0.58368 (2)	0.01438 (6)	
O2	1.08199 (9)	0.19022 (5)	0.66274 (5)	0.01998 (15)	
C2	0.77142 (12)	0.27143 (6)	0.69017 (6)	0.01592 (17)	
H2	0.8203	0.2534	0.7456	0.019*	
O3	1.04493 (9)	0.21088 (5)	0.49733 (5)	0.01909 (15)	
C3	0.62522 (12)	0.30883 (6)	0.68241 (6)	0.01519 (17)	
C3'	0.59545 (12)	0.32359 (6)	0.58690 (6)	0.01410 (17)	
C4	0.46627 (12)	0.36208 (7)	0.54150 (6)	0.01652 (18)	
H4	0.3777	0.3846	0.5737	0.020*	
C5	0.47154 (12)	0.36630 (7)	0.44789 (6)	0.01665 (18)	
C6	0.60430 (13)	0.33495 (7)	0.40057 (7)	0.01818 (19)	
H6	0.6050	0.3390	0.3363	0.022*	
C7	0.73415 (12)	0.29828 (7)	0.44561 (7)	0.01689 (18)	
H7	0.8246	0.2777	0.4136	0.020*	
C7'	0.72720 (12)	0.29276 (6)	0.53938 (6)	0.01406 (16)	
C8	0.51260 (13)	0.33196 (7)	0.75577 (7)	0.0202 (2)	
H8A	0.5623	0.3187	0.8148	0.030*	
H8B	0.4129	0.2991	0.7484	0.030*	
H8C	0.4884	0.3934	0.7525	0.030*	
C9	0.21302 (12)	0.42980 (7)	0.44164 (7)	0.01792 (18)	
H9A	0.2426	0.4792	0.4806	0.022*	
H9B	0.1713	0.3836	0.4808	0.022*	
C10	0.08711 (11)	0.45595 (6)	0.37411 (6)	0.01452 (17)	
C11	0.02533 (13)	0.53835 (7)	0.37424 (7)	0.01724 (18)	
H11	0.0674	0.5795	0.4157	0.021*	
C12	−0.09749 (14)	0.56142 (7)	0.31438 (7)	0.0217 (2)	
H12	−0.1390	0.6181	0.3148	0.026*	
C13	−0.15915 (13)	0.50175 (8)	0.25425 (7)	0.0240 (2)	
H13	−0.2447	0.5171	0.2140	0.029*	
C14	−0.09673 (13)	0.41950 (8)	0.25233 (7)	0.0222 (2)	
H14	−0.1380	0.3788	0.2101	0.027*	
C15	0.02576 (13)	0.39676 (7)	0.31194 (7)	0.01826 (18)	
H15	0.0685	0.3403	0.3105	0.022*	
C16	0.87513 (12)	0.09408 (6)	0.57724 (6)	0.01472 (17)	
C17	0.82732 (14)	0.06170 (7)	0.49366 (7)	0.0208 (2)	
H17	0.8571	0.0897	0.4392	0.025*	
C18	0.73530 (15)	−0.01231 (7)	0.49093 (7)	0.0232 (2)	
H18	0.7026	−0.0352	0.4339	0.028*	
C19	0.68993 (13)	−0.05376 (7)	0.56976 (7)	0.01902 (19)	
C20	0.73877 (14)	−0.01942 (7)	0.65257 (7)	0.0206 (2)	
H20	0.7076	−0.0469	0.7070	0.025*	
C21	0.83179 (13)	0.05392 (7)	0.65732 (7)	0.01885 (19)	
H21	0.8655	0.0765	0.7143	0.023*	
C22	0.59305 (15)	−0.13461 (8)	0.56597 (9)	0.0263 (2)	
H22A	0.5294	−0.1360	0.5095	0.040*	
H22B	0.5209	−0.1366	0.6178	0.040*	
H22C	0.6654	−0.1842	0.5681	0.040*	

### Atomic displacement parameters (Å^2^)

**Table:**

	*U*^11^	*U*^22^	*U*^33^	*U*^12^	*U*^13^	*U*^23^
N1	0.0144 (4)	0.0164 (4)	0.0135 (3)	0.0039 (3)	−0.0007 (3)	0.0004 (3)
O1	0.0149 (3)	0.0329 (4)	0.0135 (3)	0.0072 (3)	−0.0001 (3)	0.0038 (3)
S1	0.01210 (11)	0.01661 (11)	0.01444 (11)	0.00244 (8)	0.00002 (8)	0.00062 (7)
O2	0.0160 (3)	0.0249 (4)	0.0189 (3)	0.0024 (3)	−0.0046 (3)	0.0003 (3)
C2	0.0187 (4)	0.0165 (4)	0.0125 (4)	0.0018 (3)	−0.0011 (3)	0.0009 (3)
O3	0.0167 (3)	0.0226 (3)	0.0180 (3)	0.0019 (3)	0.0042 (3)	0.0027 (3)
C3	0.0174 (4)	0.0165 (4)	0.0116 (4)	0.0017 (3)	−0.0001 (3)	0.0003 (3)
C3'	0.0146 (4)	0.0158 (4)	0.0119 (4)	0.0009 (3)	0.0003 (3)	0.0002 (3)
C4	0.0149 (4)	0.0211 (4)	0.0135 (4)	0.0037 (3)	0.0005 (3)	0.0011 (3)
C5	0.0148 (4)	0.0214 (4)	0.0137 (4)	0.0021 (3)	−0.0011 (3)	0.0027 (3)
C6	0.0172 (4)	0.0249 (5)	0.0124 (4)	0.0024 (4)	0.0010 (3)	0.0021 (3)
C7	0.0154 (4)	0.0219 (4)	0.0135 (4)	0.0027 (3)	0.0021 (3)	0.0011 (3)
C7'	0.0139 (4)	0.0152 (4)	0.0130 (4)	0.0011 (3)	−0.0007 (3)	0.0010 (3)
C8	0.0221 (5)	0.0251 (5)	0.0136 (4)	0.0054 (4)	0.0027 (3)	−0.0001 (3)
C9	0.0151 (4)	0.0245 (5)	0.0142 (4)	0.0038 (4)	0.0000 (3)	0.0008 (3)
C10	0.0123 (4)	0.0173 (4)	0.0140 (4)	0.0002 (3)	0.0002 (3)	0.0024 (3)
C11	0.0183 (5)	0.0173 (4)	0.0161 (4)	0.0017 (3)	0.0008 (3)	−0.0004 (3)
C12	0.0221 (5)	0.0246 (5)	0.0186 (4)	0.0094 (4)	0.0021 (4)	0.0036 (4)
C13	0.0169 (5)	0.0378 (6)	0.0171 (4)	0.0061 (4)	−0.0024 (4)	0.0019 (4)
C14	0.0182 (5)	0.0293 (5)	0.0191 (4)	−0.0035 (4)	−0.0025 (4)	−0.0031 (4)
C15	0.0181 (5)	0.0166 (4)	0.0201 (4)	−0.0013 (3)	−0.0003 (3)	0.0003 (3)
C16	0.0146 (4)	0.0155 (4)	0.0140 (4)	0.0031 (3)	0.0007 (3)	0.0000 (3)
C17	0.0269 (5)	0.0216 (5)	0.0140 (4)	−0.0009 (4)	−0.0005 (4)	0.0006 (3)
C18	0.0292 (6)	0.0226 (5)	0.0178 (4)	−0.0022 (4)	−0.0025 (4)	−0.0025 (4)
C19	0.0169 (4)	0.0171 (4)	0.0231 (5)	0.0021 (3)	0.0019 (4)	−0.0016 (3)
C20	0.0231 (5)	0.0202 (4)	0.0187 (4)	0.0007 (4)	0.0054 (4)	0.0007 (4)
C21	0.0228 (5)	0.0196 (4)	0.0142 (4)	0.0009 (4)	0.0023 (3)	−0.0007 (3)
C22	0.0227 (5)	0.0219 (5)	0.0344 (6)	−0.0032 (4)	0.0025 (4)	−0.0030 (4)

### Geometric parameters (Å, º)

**Table:**

N1—C7'	1.4096 (13)	C17—C18	1.3855 (16)
N1—C2	1.4116 (13)	C18—C19	1.3881 (16)
N1—S1	1.6531 (9)	C19—C20	1.3889 (16)
O1—C5	1.3687 (12)	C19—C22	1.4973 (16)
O1—C9	1.4320 (13)	C20—C21	1.3824 (16)
S1—O3	1.4249 (9)	C2—H2	0.9500
S1—O2	1.4284 (9)	C4—H4	0.9500
S1—C16	1.7436 (11)	C6—H6	0.9500
C2—C3	1.3521 (14)	C7—H7	0.9500
C3—C3'	1.4454 (14)	C8—H8A	0.9800
C3—C8	1.4866 (14)	C8—H8B	0.9800
C3'—C4	1.3933 (14)	C8—H8C	0.9800
C3'—C7'	1.3955 (13)	C9—H9A	0.9900
C4—C5	1.3839 (14)	C9—H9B	0.9900
C5—C6	1.4035 (14)	C11—H11	0.9500
C6—C7	1.3831 (14)	C12—H12	0.9500
C7—C7'	1.3880 (14)	C13—H13	0.9500
C9—C10	1.4912 (14)	C14—H14	0.9500
C10—C11	1.3844 (14)	C15—H15	0.9500
C10—C15	1.3928 (14)	C17—H17	0.9500
C11—C12	1.3874 (15)	C18—H18	0.9500
C12—C13	1.3793 (17)	C20—H20	0.9500
C13—C14	1.3847 (18)	C21—H21	0.9500
C14—C15	1.3818 (15)	C22—H22A	0.9800
C16—C17	1.3852 (14)	C22—H22B	0.9800
C16—C21	1.3897 (14)	C22—H22C	0.9800
			
C7'—N1—C2	107.40 (8)	C20—C21—C16	118.84 (10)
C7'—N1—S1	124.73 (7)	C3—C2—H2	125.0
C2—N1—S1	121.67 (7)	N1—C2—H2	125.0
C5—O1—C9	115.43 (8)	C5—C4—H4	121.2
O3—S1—O2	120.40 (5)	C3'—C4—H4	121.2
O3—S1—N1	106.46 (5)	C7—C6—H6	119.3
O2—S1—N1	105.22 (5)	C5—C6—H6	119.3
O3—S1—C16	109.83 (5)	C6—C7—H7	121.3
O2—S1—C16	109.39 (5)	C7'—C7—H7	121.3
N1—S1—C16	104.19 (5)	C3—C8—H8A	109.5
C3—C2—N1	110.09 (8)	C3—C8—H8B	109.5
C2—C3—C3'	106.96 (9)	H8A—C8—H8B	109.5
C2—C3—C8	128.25 (9)	C3—C8—H8C	109.5
C3'—C3—C8	124.79 (9)	H8A—C8—H8C	109.5
C4—C3'—C7'	120.85 (9)	H8B—C8—H8C	109.5
C4—C3'—C3	131.03 (9)	O1—C9—H9A	109.9
C7'—C3'—C3	108.11 (9)	C10—C9—H9A	109.9
C5—C4—C3'	117.65 (9)	O1—C9—H9B	109.9
O1—C5—C4	124.01 (9)	C10—C9—H9B	109.9
O1—C5—C6	114.85 (9)	H9A—C9—H9B	108.3
C4—C5—C6	121.14 (9)	C10—C11—H11	119.7
C7—C6—C5	121.30 (9)	C12—C11—H11	119.7
C6—C7—C7'	117.41 (9)	C13—C12—H12	120.1
C7—C7'—C3'	121.62 (9)	C11—C12—H12	120.1
C7—C7'—N1	130.97 (9)	C12—C13—H13	119.9
C3'—C7'—N1	107.32 (8)	C14—C13—H13	119.9
O1—C9—C10	108.98 (8)	C15—C14—H14	120.1
C11—C10—C15	118.98 (9)	C13—C14—H14	120.1
C11—C10—C9	120.67 (9)	C14—C15—H15	119.7
C15—C10—C9	120.32 (9)	C10—C15—H15	119.7
C10—C11—C12	120.64 (10)	C16—C17—H17	120.6
C13—C12—C11	119.80 (10)	C18—C17—H17	120.6
C12—C13—C14	120.18 (10)	C17—C18—H18	119.3
C15—C14—C13	119.86 (10)	C19—C18—H18	119.3
C14—C15—C10	120.52 (10)	C21—C20—H20	119.3
C17—C16—C21	121.18 (10)	C19—C20—H20	119.3
C17—C16—S1	120.03 (8)	C20—C21—H21	120.6
C21—C16—S1	118.66 (8)	C16—C21—H21	120.6
C16—C17—C18	118.73 (10)	C19—C22—H22A	109.5
C17—C18—C19	121.41 (10)	C19—C22—H22B	109.5
C18—C19—C20	118.53 (10)	H22A—C22—H22B	109.5
C18—C19—C22	120.95 (10)	C19—C22—H22C	109.5
C20—C19—C22	120.52 (10)	H22A—C22—H22C	109.5
C21—C20—C19	121.32 (10)	H22B—C22—H22C	109.5
			
C7'—N1—S1—O3	42.42 (9)	S1—N1—C7'—C7	−27.34 (16)
C2—N1—S1—O3	−168.81 (8)	C2—N1—C7'—C3'	3.62 (11)
C7'—N1—S1—O2	171.26 (8)	S1—N1—C7'—C3'	156.08 (7)
C2—N1—S1—O2	−39.96 (9)	C5—O1—C9—C10	−174.12 (9)
C7'—N1—S1—C16	−73.66 (9)	O1—C9—C10—C11	−122.47 (10)
C2—N1—S1—C16	75.11 (9)	O1—C9—C10—C15	59.84 (12)
C7'—N1—C2—C3	−3.23 (11)	C15—C10—C11—C12	0.98 (15)
S1—N1—C2—C3	−156.71 (8)	C9—C10—C11—C12	−176.74 (10)
N1—C2—C3—C3'	1.52 (11)	C10—C11—C12—C13	0.17 (16)
N1—C2—C3—C8	−178.24 (10)	C11—C12—C13—C14	−1.24 (17)
C2—C3—C3'—C4	−178.08 (11)	C12—C13—C14—C15	1.14 (17)
C8—C3—C3'—C4	1.69 (18)	C13—C14—C15—C10	0.02 (16)
C2—C3—C3'—C7'	0.77 (11)	C11—C10—C15—C14	−1.07 (15)
C8—C3—C3'—C7'	−179.46 (10)	C9—C10—C15—C14	176.66 (10)
C7'—C3'—C4—C5	1.80 (15)	O3—S1—C16—C17	−17.23 (10)
C3—C3'—C4—C5	−179.47 (10)	O2—S1—C16—C17	−151.44 (9)
C9—O1—C5—C4	−1.70 (15)	N1—S1—C16—C17	96.46 (9)
C9—O1—C5—C6	178.08 (9)	O3—S1—C16—C21	166.80 (8)
C3'—C4—C5—O1	178.12 (10)	O2—S1—C16—C21	32.59 (10)
C3'—C4—C5—C6	−1.65 (16)	N1—S1—C16—C21	−79.50 (9)
O1—C5—C6—C7	−179.40 (10)	C21—C16—C17—C18	−0.29 (16)
C4—C5—C6—C7	0.38 (17)	S1—C16—C17—C18	−176.15 (9)
C5—C6—C7—C7'	0.75 (16)	C16—C17—C18—C19	0.40 (17)
C6—C7—C7'—C3'	−0.59 (15)	C17—C18—C19—C20	0.04 (17)
C6—C7—C7'—N1	−176.76 (10)	C17—C18—C19—C22	−178.85 (11)
C4—C3'—C7'—C7	−0.71 (15)	C18—C19—C20—C21	−0.61 (17)
C3—C3'—C7'—C7	−179.70 (9)	C22—C19—C20—C21	178.29 (10)
C4—C3'—C7'—N1	176.26 (9)	C19—C20—C21—C16	0.71 (16)
C3—C3'—C7'—N1	−2.73 (11)	C17—C16—C21—C20	−0.25 (16)
C2—N1—C7'—C7	−179.80 (11)	S1—C16—C21—C20	175.66 (8)
